# Lactoferrin inhibits the proliferation of IMR‑32 neuroblastoma cells even under X‑rays

**DOI:** 10.3892/mi.2023.93

**Published:** 2023-06-26

**Authors:** Shinya Kato

**Affiliations:** Radioisotope Experimental Facility, Advanced Science Research Promotion Center, Mie University, Tsu, Mie 514-8507, Japan

**Keywords:** neuroblastoma, IMR-32 cells, lactoferrin, X-rays, cell proliferation, cell membrane disruption, intracellular reactive oxygen species

## Abstract

Neuroblastoma is a typical solid tumor common in childhood. The present study investigated the inhibitory effects of lactoferrin on the proliferation of IMR-32 neuroblastoma cells, including under X-ray irradiation. In controlled *in vitro* assays, it was found that lactoferrin inhibited cell proliferation, accompanied by cell membrane disruption. Furthermore, intracellular reactive oxygen species generation increased in IMR-32 cells treated with lactoferrin, causing membrane lipid peroxidation and the leakage of lactate dehydrogenase. The IC_50_ values for cell proliferation were ~2.0 nM for doxorubicin, 2.7 mM for dibutyryl-cAMP and 45.9 µM for lactoferrin. X-ray irradiation at 1 Gy decreased cell proliferation to ~30%, which was not restored by lactoferrin. In the Fenton reaction system with iron chloride, lactoferrin increased hydroxyl radical (OH·) formation via H_2_O_2_, as confirmed by electron spin resonance spectra. On the whole, the findings of the present study indicate that lactoferrin, found abundantly in milk, may help prevent or treat neuroblastoma in infants with modest efficacy, and does not exert a protective effect against X-rays.

## Introduction

Neuroblastoma is a type of childhood cancer arising from the sympathetic ganglia of the trunk and adrenal medulla (1,2). It is the second most common solid tumor observed in childhood following leukemia and brain tumors, with an exceptionally high incidence in children <5 years of age (3-5). Half of the patients with neuroblastoma have metastases at the time of diagnosis (6). However, infants <18 months of age often have a better prognosis, and sometimes differentiate and regress spontaneously (7). These features distinguish neuroblastoma from other solid tumors.

A culture system for human neuroblastoma has been established and has been used for experiments on neuroblastoma (8,9). Lactoferrin, found in abundance in breast milk and as a protein in cow's milk, is virtually non-toxic when ingested orally and crosses the blood-brain barrier 50-fold more rapidly than transferrin (10). Lactoferrin has been found to induce neuroblastoma differentiation with the expression of β-tubulin III and neurofilaments, and to decrease survivin expression (11). Furthermore, lactoferrin recruits PI3K signaling, while both PI3K and ERK signaling are involved in inducing differentiation (11). The radioprotective effects of lactoferrin have also been studied in mice exposed to X-rays, exhibiting higher survival rates, reduced DNA damage and increased levels of superoxide dismutase following treatment with lactoferrin (12-14). These findings may be related to the mechanism of spontaneous neuroblastoma regression in infants more likely to ingest lactoferrin through the milk and indicate a potential novel application of lactoferrin; however, the underlying mechanisms remain unclear.

The author has previously examined the effects of bioactive substances on cells constituting the human brain. Recently, it was suggested that nicotine induces cellular dysfunction in human glioblastoma under lithium carbonate administration (15) and that platinum nano-colloids affect human glioblastoma cell growth in a coexisting neurotransmitter-dependent manner (16). The present study investigated the potential of lactoferrin to inhibit the proliferation of IMR-32 human neuroblastoma cells compared to doxorubicin and dibutyryl cyclic AMP (db-cAMP), including under X-ray irradiation conditions.

## Materials and methods

### Cells and cell culture

The IMR-32 human neuroblastoma cell line was obtained from the JCRB cell bank (cat. no. JCRB9050). The IMR-32 is a fibroblast-like cell line established by W.W. Nichols in 1970, which was obtained during exploratory surgery from an abdominal mass of a 13-month-old boy (8). The proto-oncogene N-*myc* (MYCN), a genetic signature of neuroblastoma, is amplified in IMR-32 cells (17,18). The IMR-32 cells were cultured in Eagle's minimum essential medium (E-MEM) supplemented with non-essential amino acids (056-08385, FUJIFILM Wako Pure Chemical Corp.), L-glutamine (073-05391, FUJIFILM Wako Pure Chemical Corp.), 10% fetal bovine serum (S-FBS-NL-015, Serana Europe GmbH) and penicillin-streptomycin-amphotericin B suspension (161-23181, FUJIFILM Wako Pure Chemical Corp.) at 37˚C with 5% CO_2_.

### Lactoferrin

Lactoferrin from bovine milk (123-04124, FUJIFILM Wako Pure Chemical Corp.) has a molecular weight of ~83,000 kDa and an iron saturation of 3.6-25.0%. Lactoferrin was dissolved in PBS (-) of pH 7.4, phosphate-buffered saline without Ca and Mg (164-23551, FUJIFILM Wako Pure Chemical Corp.), at a concentration of 1 mg/ml, and subjected to light scattering measurement using a zeta-potential and particle size analyzer (ELSZneo, Otsuka Electronics Co. Ltd.), and resulted in electrophoretic mobility of -2.71±0.51x10^-5^ cm^2^/Vsec, a particle size of 14.3±0.1 nm and a molecular weight of 1.669x10^5^, suggesting that lactoferrin exists as an aggregate in the PBS (-) solution.

### Cell proliferation assay

The 2-(2-methoxy-4-nitrophenyl)-3-(4-nitrophenyl)-5-(2,4-disulfophenyl)-2H-tetrazolium (WST-8) assay with a water-soluble tetrazolium salt was employed to assess cell proliferation (19). The IMR-32 cells were seeded at 3,000 cells/well in a 96-well culture plate (Sumitomo Bakelite Co., Ltd.) as n=5 and pre-incubated for 24 h at 37˚C with 5% CO_2_. Lactoferrin was added to each well at 0-120 µM. Following a 1-day incubation, the cells were exposed to X-rays at 1 Gy (CAX-150-20; Chubu Medical Co., Ltd.; 150 kV-20 mA, 1 mm Al + 0.1 mm Cu filters, 0.60 Gy/min) and incubated for 6 days at 37˚C with 5% CO_2_. The medium was then replaced with 5% of WST-8 solution (cat. no. 347-07621, Dojindo Laboratories, Inc.) diluted with E-MEM and incubated for 1.5 h at 37˚C with 5% CO_2_. Subsequently, the absorbance was measured at λ=450 nm with a multi-spectrophotometer (Viento, Dainippon Sumitomo Pharma, Co. Ltd.). The amount of formed formazan is proportional to the number of viable cells, as intracellular mitochondrial dehydrogenase reduces WST-8 to yellowish-orange formazan (19). In addition, was conducted in a single administration with db-cAMP at 0-4.8 nM (cat. no. sc-201567, Santa Cruz Biotechnology, Inc.), an inducer of neuroblastoma cell differentiation, and doxorubicin hydrochloride at 0-3.84 mM (040-21521, FUJIFILM Wako Pure Chemical Corp.), an anticancer drug. The doubling time of the IMR-32 cells was ~36 h, and the cells were in a logarithmic growth phase during 7 days of culture. Cell morphology was observed under a phase contrast microscope (CKX-53, Olympus Corp.) at a magnification of x200. Cell proliferation was evaluated by repeating the experiment five times, and the IC_50_ value for each reagent was determined. The IC_50_ values were estimated by plotting a series of dose-response data with the logarithm of the dose and using a fitted straight line.

### Membrane lipid peroxidation and leakage of lactate dehydrogenase

Cell membrane disruption was evaluated by membrane lipid peroxidation and lactate dehydrogenase leakage. The IMR-32 cells were seeded at 5,000 cells/well in a 96-well culture plate as n=5 and pre-incubated for 24 h at 37˚C with 5% CO_2_. Lactoferrin was added to each well at 0-30 µM. Following a 1-day incubation, the cells were exposed to X-rays at 1 Gy and incubated for 24 h at 37˚C with 5% CO_2_. The cell culture medium was then replaced with 1 µmol/l N-(4-diphenylphosphinophenyl)-N'-(3,6,9,12-tetraoxatridecyl)perylene-3,4,9,10-tetracarboxydiimide (Liperfluo, Dojindo Laboratories, Inc.) for the detection of lipid hydroperoxides (20). Following 1.5 h of incubation at room temperature in the dark, the fluorescence intensity, proportional to lipid peroxide in membrane lipids, was measured at Ex/Em=485 nm/535 nm using a microplate reader (TriStar LB941, Berthold Technologies GmbH & Co. KG). On the other hand, the cell culture medium was replaced with E-MEM containing water-soluble formazan of the cytotoxicity LDH-assay kit (Dojindo Laboratories, Inc.). Following 0.5 h of incubation at room temperature in the dark, the absorbance at 490 nm, proportional to lactate dehydrogenase leakage (21), was measured using the multi-spectrophotometer (Viento, Dainippon Sumitomo Pharma, Co. Ltd.).

### Levels of apoptosis-mediating caspase-3/7

The IMR-32 cells were seeded at 5,000 cells/well in a 96-well culture plate (SPL Life Sciences Co., Ltd.) as n=5 and pre-incubated for 24 h at 37˚C with 5% CO_2_. Lactoferrin was added to each well at 0-120 µM. Following a 1-day incubation, the cells were exposed to X-rays at 1 Gy and incubated for 24 h at 37˚C with 5% CO_2_. The cell culture medium was then replaced with E-MEM containing the Caspase-Glo 3/7 assay system (Promega Corp.). Following 0.5-h of incubation at room temperature in the dark, the luminescence intensity, proportional to caspase-3/7 activity, was measured using the microplate reader (TriStar LB941, Berthold Technologies GmbH & Co. KG).

### Measurement of intracellular reactive oxygen species

The nitroblue tetrazolium (NBT) reduction method was employed to assess the production of superoxide anion radicals (O_2_·^-^) in cells (22,23). The IMR-32 cells were seeded at 12,000 cells/well in a 96-well culture plate as n=5 and pre-incubated for 24 h at 37˚C with 5% CO_2_. Lactoferrin was added to each well at 0-30 µM. Following a 1-day incubation, the medium was replaced with 0.2% NBT (Tokyo Chemical Industry, Co., Ltd.)-containing medium filtered <0.22 µm. The cells were exposed to X-rays at 1 Gy and incubated at 37˚C with 5% CO_2_. Following a 3-h incubation, the absorbance of NBT-formazan was measured at l=620 nm using the multi-spectrophotometer (Viento) and cell morphology was observed using a phase contrast microscope (CKX-53, Olympus Corp.) at a magnification of x200.

### Intracellular uptake of lactoferrin

The intracellular uptake of lactoferrin was assessed by immunostaining with a goat anti-bovine lactoferrin antibody. The IMR-32 cells were seeded at 36,000 cells/well in a chamber slide (Nalge Nunc International Corp.) and pre-incubated for 3 days at 37˚C with 5% CO_2_. Lactoferrin was added to each well at a dose of 1.2 µM with no cytotoxicity. The cells were rinsed with E-MEM following incubation for 0.2, 6 and 24 h at 37˚C. Subsequently, 4% paraformaldehyde phosphate buffer solution at pH 7.4 (FUJIFILM Wako Pure Chemical Corp.) was added, and the cells were allowed to stand for 15 min at room temperature. The cells were then washed with PBS(-) (FUJIFILM Wako Pure Chemical Corp.) and permeabilized with 0.1% Triton X-100 (FUJIFILM Wako Pure Chemical Corp.) for 5 min on ice. Blocking was carried out with 2% rabbit serum (Cedarlane Laboratories, Inc.) in PBS(-) for 30 min at room temperature, and the cells were then allowed to react with a goat anti-bovine lactoferrin antibody (1:200 dilution; cat. no. A10-126, Bethyl Laboratories, Inc.) as a primary antibody overnight at 4˚C. After washing with PBS(-), an FITC-conjugated rabbit anti-goat IgG antibody (1:200 dilution; cat. no. SA00003-4, Proteintech Group, Inc.) was added as a secondary antibody to react for 1 h at room temperature. The cell nuclei were then stained with DAPI (D523, 1:500 dilution, Dojindo Laboratories, Inc.) for 15 min at room temperature, and the cells were observed under a phase contrast fluorescence microscope (CKX-53) at Ex/Em: 330-385 nm/420 nm and 460-495 nm/510 nm, and a magnification of x400.

### Measurement of hydroxyl radical (OH·) formation using electron spin resonance spectroscopy (ESR)

The formation of hydroxyl radicals (OH·) in the Fenton reaction system with Fe_2_Cl_2_/H_2_O_2_ was measured using ESR with a spin trap method with 5,5-dimethyl-1-pyrroline N-oxide (DMPO, MilliporeSigma). A hydrogen peroxide solution (35%, Nacalai Tesque, Inc.) of 0.5 ml was added to a glass vial, followed by a drop of 2.5% iron chloride (FeCl_2_)·4H_2_O solution (Nacalai Tesque, Inc.), 0.5 ml of lactoferrin solution of 120 µM. Subsequently, 0.01 g DMPO was added, which required ~30 sec. The mixed solution was then filled into a flat quartz cell and measured using a ESR spectrometer (JES-FA200, JEOL Ltd.). The ESR spectra were obtained at a microwave power level of 0.4 and 100 kHz filed modulation at room temperature. The magnetic field was calibrated with the well-known splitting constants of Mn^2+^ in MgO.

### Statistical analysis

Cell proliferation, membrane lipid peroxidation, the leakage of lactate dehydrogenase and the levels of caspase-3/7 activity are expressed as the mean ± SD, n=5. The data were analyzed using one-way ANOVA followed by Dunnett's test with KaleidaGraph 4.5J software (HULINKS Inc.). A value of P<0.05 was considered to indicate a statistically significant difference.

## Results

### Proliferation of IMR-32 neuroblastoma cells

**Figure 1:**
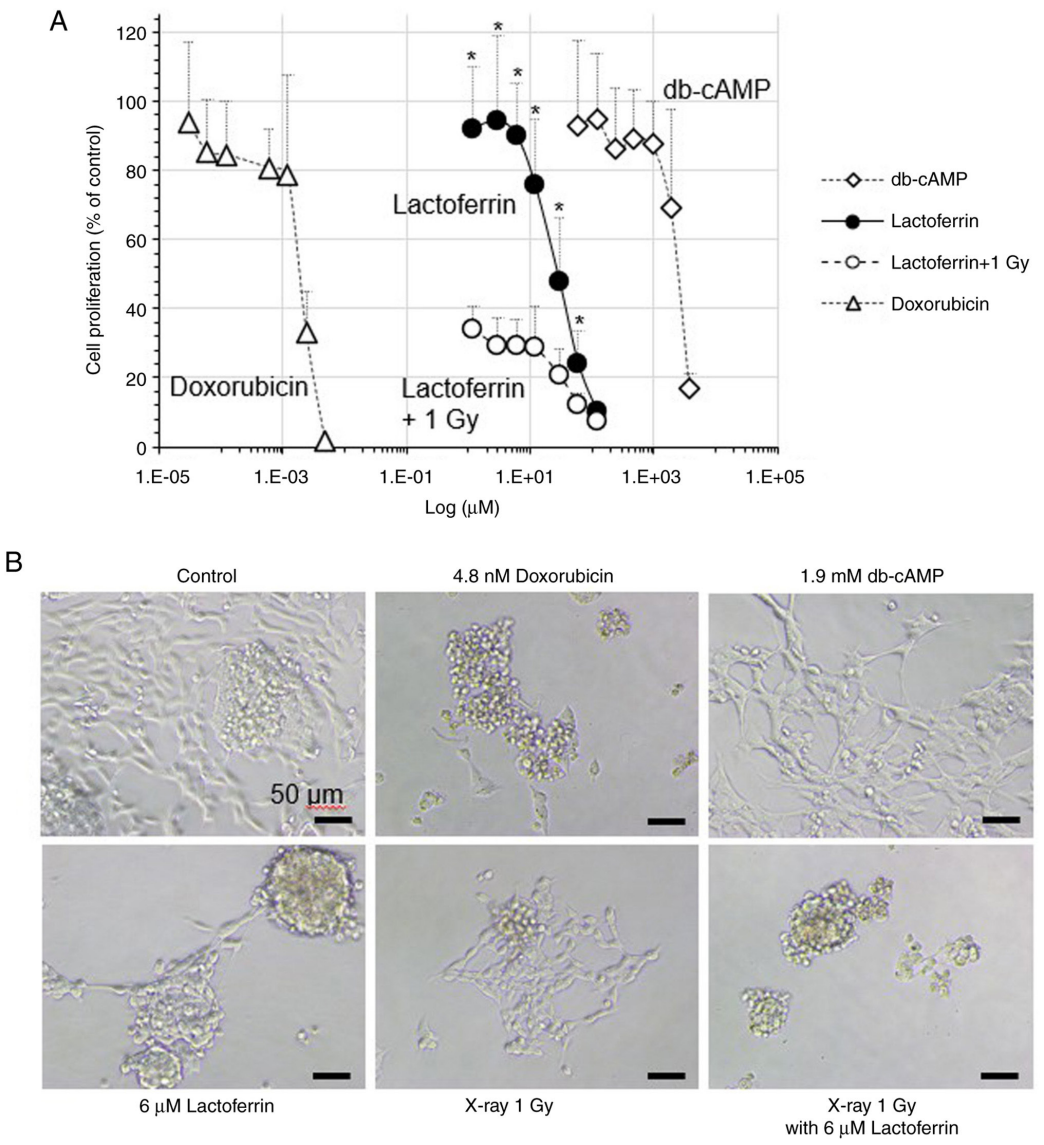
Proliferation of IMR-32 human neuroblastoma cells. (A) The IMR-32 cells were treated with 0-4.8 nM doxorubicin, 0-3.84 mM db-cAMP, or 0-120 µM lactoferrin and exposed to X-rays at 1 Gy. Following a 6-day incubation, cell proliferation was measured using mitochondrial dehydrogenase-reduced formazan-based WST-8 assay. Data are presented as the mean ± SD, n=5. ^*^P<0.05, lactoferrin vs. lactoferrin +1 Gy. (B) Cell morphology was observed under a phase contrast microscope. Magnification, x200; scale bar, 50 µm. db-cAMP, dibutyryl cyclic AMP.

In the IMR-32 cells treated with 1.2-120 µM lactoferrin, the cell proliferation rate (% of control) decreased from 92.8 to 10.3% in a concentration-dependent manner (Fig. 1). The cell proliferation rate decreased to 33.9% following X-ray irradiation at 1 Gy, and treatment with 120 µM lactoferrin prior to X-ray exposure led to a cell proliferation rate of 7.2%, indicating that lactoferrin has no radioprotective effect. When doxorubicin, an anticancer drug, was administered at concentrations of 0.03-4.8 nM, cell proliferation decreased from 93.7 to 1.5%. In addition, db-cAMP, a differentiation inducer, at concentrations of 60-3.84 mM, reduced cell proliferation from 92.8 to 17.1%. The IC_50_ values for cell proliferation were ~2.0 nM for doxorubicin, 2.7 mM for db-cAMP and 45.9 µM for lactoferrin. IC_50_ values were calculated from experimental data repeated five times. The differentiation inducer db-cAMP induced neurite outgrowth, whereas lactoferrin treatment did not increase neurite outgrowth (Fig. 1).

### Intracellular reactive oxygen species

**Figure 2:**
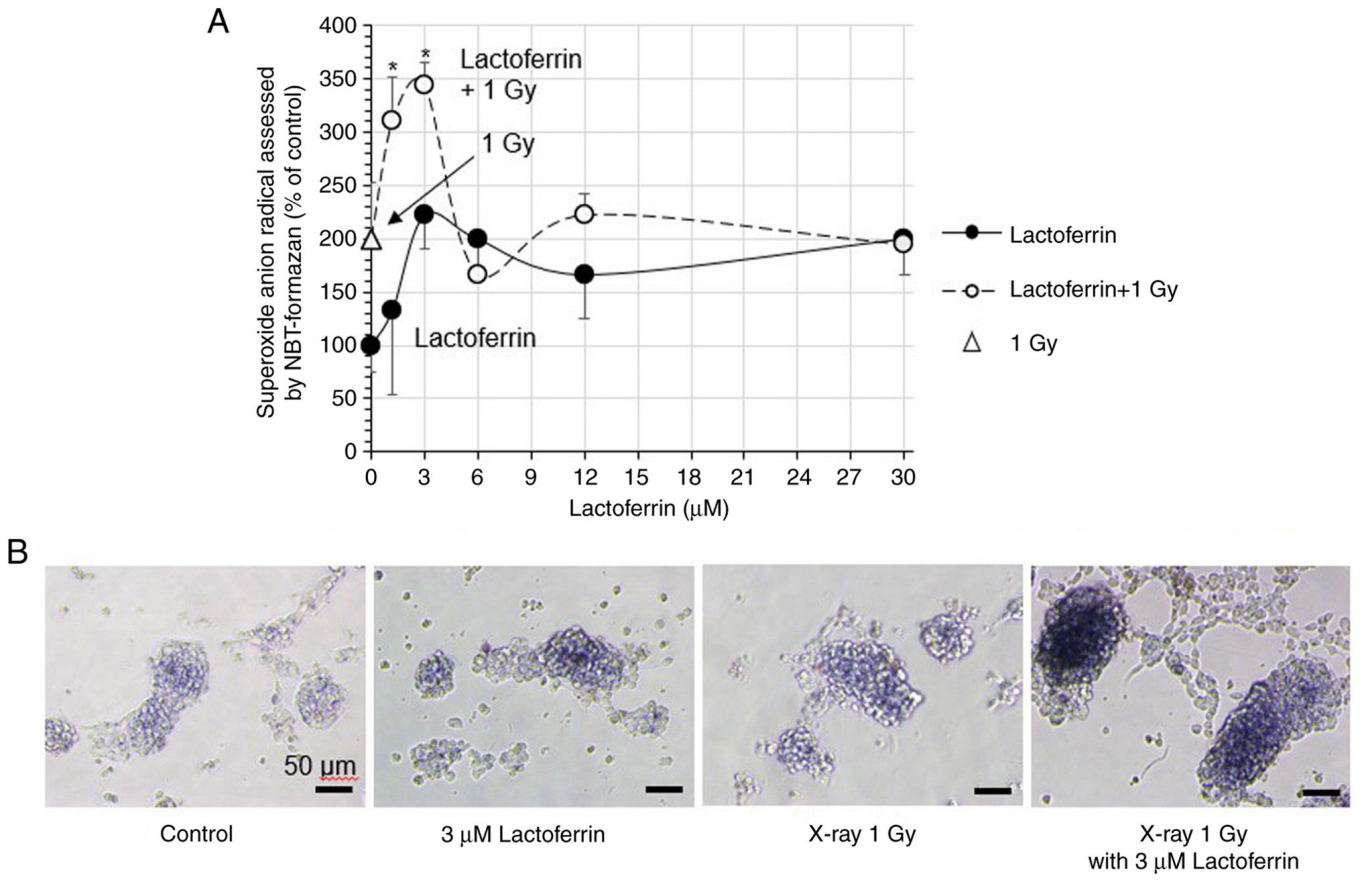
Intracellular reactive oxygen species generation in human neuroblastoma IMR-32 cells. (A) The IMR-32 cells were treated with 0-30 µM lactoferrin, replaced with 0.2% NBT-containing medium, and exposed to X-rays at 1 Gy. Following a 1.5-h incubation, the superoxide anion radicals (O_2_·-) were measured using a microplate reader. Data are presented as the mean ± SD, n=5. ^*^P<0.05, lactoferrin vs. lactoferrin +1 Gy. (B) Cell morphology was observed under a phase contrast microscope. Magnification, x200; scale bar, 50 µm.

In the IMR-32 cells treated with 1.2-30 µM lactoferrin, superoxide anion radicals (O_2_·^-^; % of control) increased in a concentration-dependent manner to 194.4%, with a peak of 222.2% at 3 µM (Fig. 2A). X-ray irradiation at 1 Gy increased superoxide anion radicals to 200.0%, and treatment with 3 µM lactoferrin increased them to a peak of 344.4%. Micrographs of the cells exhibited a blue color of NBT formazan corresponding to the formation of superoxide anion radicals (Fig. 2B).

### Intracellular uptake of lactoferrin

**Figure 3:**
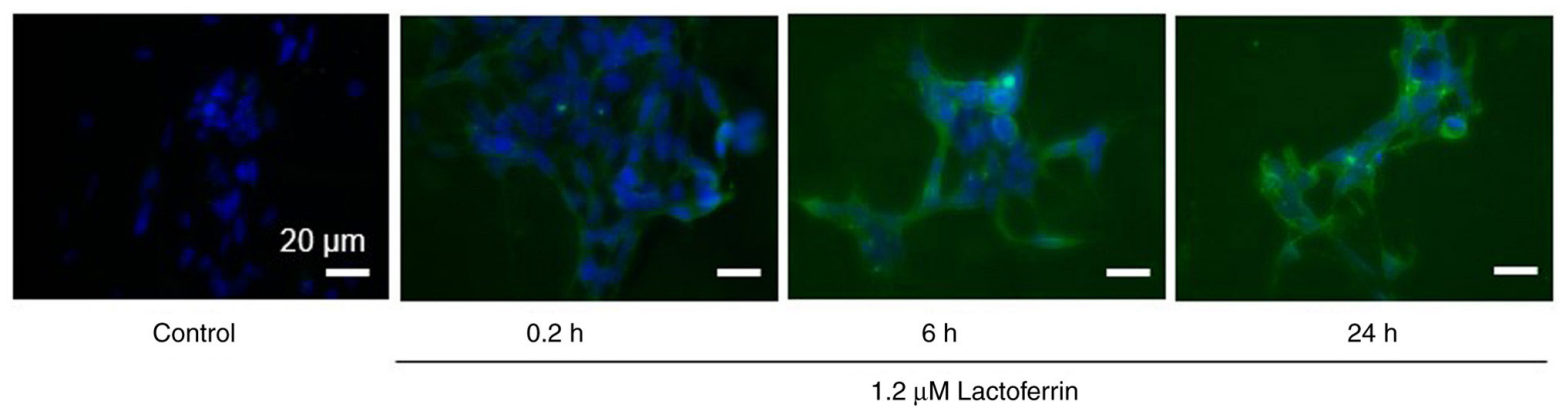
Intracellular uptake of lactoferrin by IMR-32 human neuroblastoma cells. The IMR-32 cells were treated with 1.2 µM lactoferrin at a non-cytotoxic concentration and incubated at 37˚C, 5% CO_2_. Following 0.2, 6 and 24 h of incubation, the cells were immunostained with a goat anti-bovine lactoferrin antibody and observed under a phase contrast fluorescence microscope. Cell nuclei were stained with DAPI. Magnification, x400; scale bar, 20 µm.

Fluorescence microscopy images of the IMR-32 cells treated with 1.2 µM lactoferrin for 0.2, 6 and 24 h revealed that lactoferrin was gradually incorporated into the cells over time (Fig. 3).

### Membrane lipid peroxidation and leakage of lactate dehydrogenase

**Figure 4:**
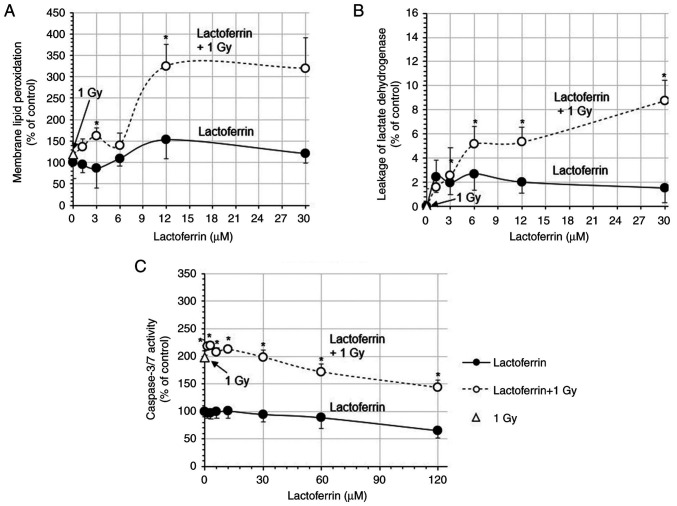
Cell membrane disruption and caspase-3/7 activity in human neuroblastoma IMR-32 cells. The IMR-32 cells were treated with 0-30 or 120 µM lactoferrin and exposed to X-rays at 1 Gy. Following a 24-h incubation, cell membrane disruption was assessed by fluorescence measurements of (A) membrane lipid peroxidation, (B) lactate dehydrogenase leakage, and (C) apoptosis-mediated caspase-3/7 levels assessed using luminescence measurements. Data are presented as the mean ± SD, n=5. ^*^P<0.05, lactoferrin vs. lactoferrin +1 Gy.

In the IMR-32 cells treated with 1.2-30 µM lactoferrin, membrane lipid peroxidation (% of control) gradually increased to 121.2% (Fig. 4A). X-ray irradiation at 1 Gy increased membrane lipid peroxidation to 118.8%, which increased to 324.8% with 12 µM lactoferrin (Fig. 4A). The leakage of lactate dehydrogenase also gradually increased by 1.5-2.7%. Although X-rays at 1 Gy did not increase it, following X-ray irradiation at 1 Gy with lactoferrin, the leakage of lactate dehydrogenase was increased by 5.1% at 6 µM and 8.8% at 30 µM, indicating a significant synergic effect of X-rays and lactoferrin (Fig. 4B).

The apoptosis-mediating caspase-3/7 activity decreased gradually from 97.4 to 65.5% in cells treated with 1.2-120 µM lactoferrin, but increased rapidly to 198.0% following X-ray irradiation at 1 Gy. In the cells treated with lactoferrin and X-rays at 1 Gy, caspase-3/7 activity decreased gradually from 218.7 to 143.2% (Fig. 4C).

### Hydroxyl radical (OH·) formation measured using ESR

**Figure 5:**
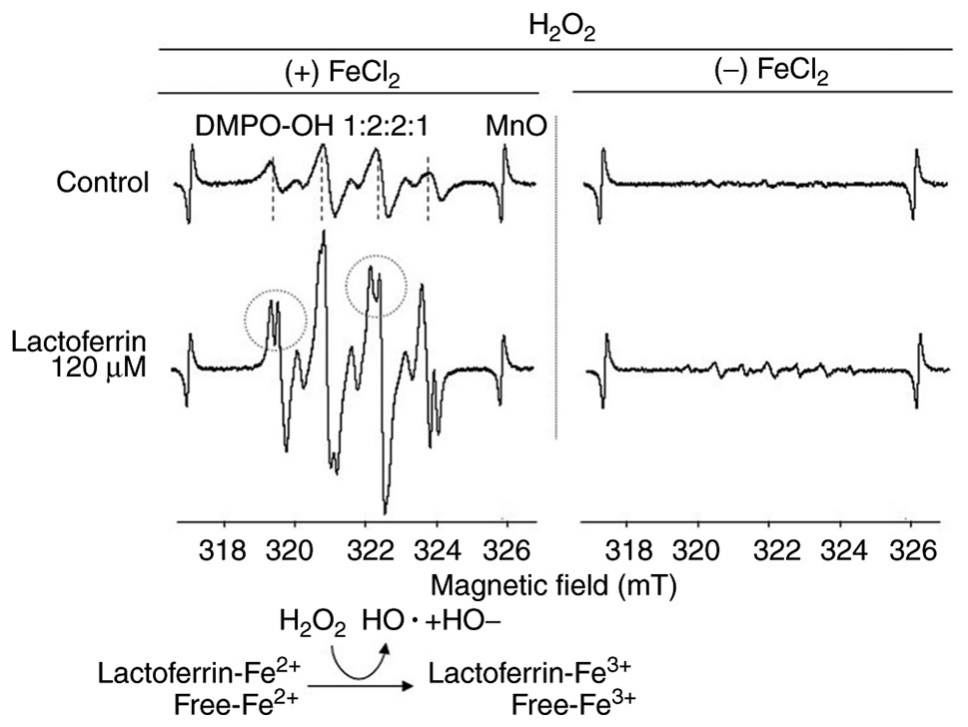
Hydroxyl radical (OH·) formation in the Fenton reaction system with lactoferrin. Lactoferrin at 120 µM was added to the Fenton reaction system, and DMPO-trapped OH· signals were measured by electron spin resonance, which was performed with (+) or without (-) the addition of FeCl_2_. The dotted circle indicates a split DMPO-OH· signal, suggesting a mechanism involving lactoferrin and iron ions (Fe^2+^, Fe^3+^). DMPP, 5,5-dimethyl-1-pyrroline N-oxide; FeCl_2_, iron chloride.

When lactoferrin was added in the reaction system without iron chloride, the levels of hydroxyl radicals (OH·) did not markedly increase. However, in the Fenton reaction system with iron chloride, the coexistence of lactoferrin resulted in a considerable increase in the formation of hydroxyl radicals (OH·) compared with the control, which split into two peaks (Fig. 5).

## Discussion

The present study investigated the inhibition of the proliferation of IMR-32 human neuroblastoma cells by lactoferrin, including under X-ray irradiation. In IMR-32 human neuroblastoma cells, a concentration-dependent decrease in cell proliferation was observed. X-ray irradiation at 1 Gy reduced cell proliferation to ~30% and cell proliferation was not restored by lactoferrin treatment prior to X-ray irradiation. The IC_50_ values were ~2.0 nM for doxorubicin, 2.7 mM for db-cAMP and 45.9 µM for lactoferrin. Neurite outgrowth was observed with db-cAMP, although no increase in neurite outgrowth was observed with lactoferrin treatment. This may be due to the fact that the medium containing 10% FBS was unfavorable for inducing differentiation. Thus, lactoferrin inhibited the growth of neuroblastoma cells, although not as markedly as the anticancer drug, doxorubicin.

Lactoferrin increased intracellular superoxide anion radicals (O_2_·^-^), further augmented by X-ray irradiation at 1 Gy with lactoferrin, reaching a peak at 1.2-3 µM. Membrane lipid peroxidation was also increased by X-rays with lactoferrin, peaking at a relatively low concentration of 12 µM. In addition, cellular immunostaining revealed that lactoferrin was gradually taken into the cells over a period of 24 h following administration. Based on previous studies, the lactoferrin receptor is highly expressed on the apical surface of respiratory epithelial cells, as well as brain endothelial cells and neurons (24,25). Although lactoferrin has a large molecular weight, the cellular uptake of lactoferrin nanoparticles has been observed in SH-SY5Y neuroblastoma cells (26). Recently, the radioprotective effects of lactoferrin have been reported. Wei *et al* (14) reported that lactoferrin prolonged the survival rate of mice exposed to 8 Gy of X-rays, which was attributed to the suppression of intestinal injury through the reduction of inflammatory cytokines and the downregulation of NF-κB. Feng *et al* (12) reported that in the hepatic tissue of mice exposed to 7 Gy X-rays, treatment with lactoferrin increased the levels of superoxide dismutase and decreased those of malondialdehyde, suggesting that lactoferrin may prevent radiation damage in patients undergoing radiotherapy. In contrast to these reports, the results of the present study indicated that intracellularly incorporated lactoferrin did not exert an antioxidant effect, but promoted intracellular oxidation. Moreover, lactoferrin slightly increased the leakage of lactate dehydrogenase from cells, which was significantly increased by X-rays in combination with lactoferrin. The bovine lactoferrin used in the experiments in the present study had an electrophoretic mobility of -2.71x10^-5^ cm^2^/Vsec, which was slightly negatively charged and may have caused damage by binding to the cell membrane or being taken up into the cell. On the other hand, the levels of apoptosis-mediating caspase-3/7 activity were significantly increased by X-ray irradiation at 1 Gy, but not by lactoferrin, suggesting that lactoferrin does not actively induce the apoptosis of IMR-32 cells. These results indicate that the mechanism of cell growth inhibition by lactoferrin involves membrane damage rather than apoptosis in the cells.

ESR measurements revealed that the hydroxyl radical signal increased and split in two upon the addition of lactoferrin. The two split peaks indicate multiple environments for forming hydroxyl radicals (OH·), suggesting the involvement of ferrous iron trapped in lactoferrin and free ferrous ions in the reaction system. In the ESR measurements of lactoferrin-mediated radicals, Nishimura *et al* (13) demonstrated that lactoferrin scavenged superoxide anion radicals (O_2_·^-^) generated in the hypoxanthine-xanthine oxidase system and hydroxyl radicals (OH·) generated in the Cu(en)_2_ or H_2_O_2_/ultraviolet-ray system. On the other hand, hydroxyl radical production, measured using ESR, has been demonstrated to be produced by a Fenton-type Haber-Weiss reaction catalyzed by lactoferrin (27). It has also been reported that oxidation of Fe^2+^ is accelerated in the presence of lactoferrin and that Fe^2+^ and lactoferrin produces ·OH via an H_2_O_2_ intermediate with toxicity to microorganisms (28). Of note, the present study focused on a reaction system where lactoferrin and ferrous ion coexist. The results in the Fenton reaction system with Fe_2_Cl_2_/H_2_O_2_ indicated that lactoferrin increased the hydroxyl radical (OH·) formation via H_2_O_2_. Bovine lactoferrin, a glycoprotein with two symmetric lobes, can bind one ferric ion per lobe and prevent the echovirus-induced cytopathic effect (29). Chung and Raymond (30) reported that apoproteins prefer an ‘open’ conformation in which the iron-binding site is close to the protein surface and exposed to the surrounding solution, whereas lactoferrin becomes a closed, stable form when the iron is bound and is less likely to release iron than transferrin. It is known that bovine lactoferrin and natural human lactoferrin have similar three-dimensional structures, with human lactoferrin consisting of 691 and bovine lactoferrin composed of 689 amino acids (31). The iron saturation of bovine lactoferrin is 15-20%, and that of natural human lactoferrin is ~10% (32). The bovine lactoferrin used in the present study has an iron saturation of 3.6-25.0%, indicating that it has extra capacity to capture iron ions. Generally, it has been considered that when lactoferrin is administered to cells, lactoferrin removes iron, thereby decreasing oxidative stress in the cells (33,34); however, this was not the case in the present study. It was hypothesized that when lactoferrin takes up ferrous irons, the ferrous ions are immediately oxidized and stabilized as ferric irons, and along with that oxidation process, reactive oxygen species are generated in cells.

In the present study, the author first tried DCFH-DA to detect a wide range of reactive oxygen species in cells; however, as there was a problem with cells peeling off, the author switched to the NBT method. Since superoxide anion radicals are also critical intracellular reactive oxygen species, this has been discussed as much as possible with the data in the present study. In the future, the author would also like to examine how lactoferrin treatment affects drug-resistant neuroblastoma cells, such as SK-N-Be2c and KCNR.

In conclusion, the present study demonstrated that lactoferrin inhibited the proliferation of neuroblastoma cells even under X-rays, accompanied by cell membrane disruption. In the Fenton reaction system with Fe_2_Cl_2_/H_2_O_2_, lactoferrin increased hydroxyl radical (OH·) formation via H_2_O_2_, as confirmed by ESR spectra. Lactoferrin, which is found abundantly in milk and a food component in dairy products, may help to prevent or treat neuroblastoma in infants with modest efficacy, and it did not exert a protective effect against X-rays.

## Data Availability

The datasets used and analyzed during the current study are available from the corresponding author upon reasonable request.
